# Liquid Low-Level Radioactive Waste Treatment Using an Electrodialysis Process

**DOI:** 10.3390/membranes11050324

**Published:** 2021-04-28

**Authors:** Agnieszka Miśkiewicz, Agnieszka Nowak, Jędrzej Pałka, Grażyna Zakrzewska-Kołtuniewicz

**Affiliations:** Institute of Nuclear Chemistry and Technology, Dorodna 16, 03-195 Warsaw, Poland; agnowak95@gmail.com (A.N.); jedrzej.palka.1996@wp.pl (J.P.); g.zakrzewska@ichtj.waw.pl (G.Z.-K.)

**Keywords:** membrane process, electrodialysis, radioactive wastes, decontamination

## Abstract

In this work, the possibility of using electrodialysis for the treatment of liquid low-level radioactive waste was investigated. The first aim of the research was to evaluate the influence of the process parameters on the treatment of model solutions with different compositions. Subsequent experimental tests were conducted using solutions containing selected radionuclides (^60^Co and ^137^Cs), which are potential contaminants of effluents from nuclear power plants, as well as components often found in waste generated from industrial and medical radioisotope applications. The results of the experiments performed on real radioactive waste confirmed that electrodialysis was a suitable method for the treatment of such effluents because it ensured high levels of desalination and rates of decontamination. The most important parameters impacting the process were the applied voltage and electrical current. Moreover, this research shows that the application of the ED process enables the separation of non-ionic organic contaminants of LLW, which are unfavorable in further stages of waste predisposal.

## 1. Introduction

Many activities related to nuclear energy production generate radioactive waste, although such waste may also be produced following the medical or industrial use of radioisotopes. In general, radioactive waste differs in terms of form, radioactivity concentration, and type of contamination, depending on its origin. It can exist in a solid, liquid, or gaseous state, and the concentration levels of radioactivity can range from very high (e.g., in spent fuel and waste from its reprocessing) to very low (e.g., from the use of radioisotopes in laboratories, hospitals, and other industries) [1]. 

Low-level radioactive liquid wastes (LLWs) from industrial and medical applications typically contain short-lived radionuclides (i.e., half-life < 30 years) and very rarely contain trace concentrations of long-lived radionuclides. Such wastes may also include small amounts of spent acids and bases, analytical solutions, scintillation cocktails, decontamination and cleaning solutions, or waste oils. Overall, LLWs are very complex solutions, and without appropriate treatment they are a hazard to human health and the surrounding environment. Therefore, because of their potential detriments, such wastes cannot be directly discharged into the environment; they must be processed to reduce their volume and allow for further stabilization that makes them more suitable for long-term disposal. Various methods have been employed for the treatment of radioactive waste, including chemical precipitation, ion exchange, thermal evaporation, and solvent extraction [2]. Despite their demonstrated performance, these methods have several drawbacks, such as their high energy consumption and the formation of secondary waste (e.g., spent ion-exchange resins, various types of adsorbents, regeneration solutions, or sludge from sediment tanks). The range of methods that can be used for LLW treatment are constantly expanding, and new approaches, including electrochemical and biotechnological methods and membrane processes, have been adopted for this purpose. In particular, membrane processes have been successfully employed for liquid radioactive waste treatment owing to a number of advantages, including their high decontamination factors, large volume reduction, and low energy consumption [3,4]. Moreover, membrane systems are flexible and it is not difficult to combine them with other treatment methods. It has also been demonstrated that membrane processes offer improved treatment capabilities when applied in combination with other methods, especially in cases where conventional methods alone are not efficient or effective enough [3]. There are many examples of membrane implementation in radioactive waste processing technology [5,6,7]. Many additional strategies for implementing membrane processes in radioactive liquid waste processing plants have been investigated [8,9,10,11]. 

To our knowledge, there are a limited number of reports describing electrical processes such as electrodialysis (ED) for the treatment of radioactive liquid waste. Electrodialysis is based on ion-exchange membranes that use electrochemical potential as the driving force [12]. The main advantages of ED used for liquid waste treatment include its low-pressure operation, low lifecycle costs, and easy maintenance. Although the electrodialysis process is high in capital cost, it provides a product with a higher quality and is more environmentally friendly than other mass separation techniques [13]. Electrodialysis has been utilized primarily for the production of potable water from brackish water sources [14]. However, there are some reports that describe the application of ED in the nuclear field—namely, the separation of molybdenum, carbonate, and bicarbonate ions from liquid waste containing uranium in the uranium ore leaching process [15]. The results of these studies confirmed the high retention of carbonate ions and selective separation of molybdenum from uranium. Electrodialysis combined with diffusion dialysis was tested for the separation of ^137^Cs and ^90^Sr from concentrated nitric acid solutions [16]. Diffusion dialysis was conducted in a two-chamber system wherein the anion-exchange membrane and the reduction in solution acidity were involved in the first step of the proposed radioactive waste treatment scheme. Electrodialysis using novel anion-exchange membranes was also applied for the separation of ^125^I and ^36^Cl ions [17]. Prepared paper membranes containing trimethylhydroxylpropylammonium groups exhibited a high selectivity for iodide ions over chloride ions, thereby supporting their use for iodide removal from radioactive waste. 

The aim of the present work was to evaluate the possibility of using a batch electrodialysis process to treat low-level radioactive liquid waste from the use of radioisotopes in research, medicine, and industry. The waste was collected by the Radioactive Waste Management Plant (RWMP) in Swierk, Poland, and processed using conventional methods, such as evaporation or chemical precipitation. In addition to high concentrations of inorganic salts, this type of waste commonly contains organic compounds derived, for example, from decontamination of laboratory or equipment. The presence of organic substances affects later stages of radioactive waste processing and the general safety of its final disposal. Therefore, it is advisable to separate such compounds before the next treatment stages. Herein, we demonstrate that the ED process represents a promising technique to achieve this goal.

## 2. Materials and Methods

### 2.1. Chemicals

All chemicals used in the work were of analytical grade purity. Salts and citric acid used in experiments were obtained from CHEMPUR, Poland. Triton X-102 (polyethylene glycol octylphenyl ether, MW = 757 g/mol) was purchased from Sigma Aldrich (St. Louis, MO, USA). Deionized water was used in all experiments. The carrier-free radionuclides of cesium-137 (radioactive half-life, t_1/2_ = 30.07 y; energy of emitted radiation, E_γ_ = 661.7 keV) and cobalt-60 (t_1/2_ = 5.3 y; E_γ_ = 1.17 and 1.33 MeV) were supplied by POLATOM (Otwock-Swierk, Poland) as certificated standard solutions. 

### 2.2. Membrane Installation

**Figure 1:**
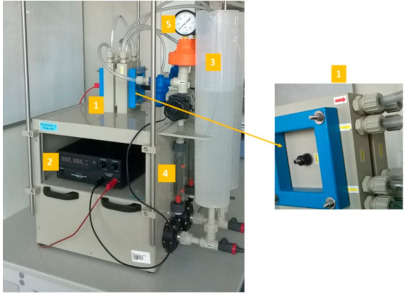
Photo of the installation for the electrodialysis process: 1—ED cell; 2—power supply; 3—solution tanks; 4—rotameters; 5—manometer.

The experiments were conducted with the ED installation, BED 1-2 Compact (PCCell GmbH, Heusweiler, Germany) equipped with an electrodialysis cell (type 64002), as illustrated in Figure 1.

**Figure 2:**
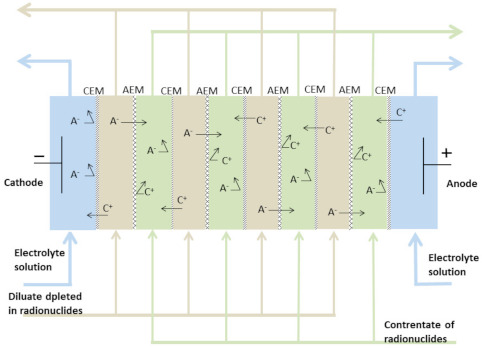
Scheme of membranes configuration in the ED cell.

The ED installation contained an electrodialysis stack of ten anion-exchange membranes (PC-SA) and nine cation-exchange membranes (PC-SK), whose parameters are presented in Table 1. A scheme of the membrane configuration in a processing cell is illustrated in Figure 2. Overall, the ED installation consisted of an electrode solution tank (volume = 2 dm^3^), external cylinders for the diluate and concentrate (volume = 2 dm^3^ each), and a power supply (HCS-3202, Manson, Kwai Chung, N.T, Hong Kong). The concentrate, diluate, and electrode solutions circulated in three independent circuits, each of which was equipped with a separate pump and rotameter. The mean velocities of the diluate and concentrate were maintained at 0.16 cm/s.

### 2.3. Methods

At the beginning of the ED experiments, the solution to be purified was placed in the diluate and concentrate cylinders. A solution of 0.1 M Na_2_SO_4_ was used as the electrolyte and the concentrate and diluate solutions were circulated through the ED installation. 

**Figure 3:**
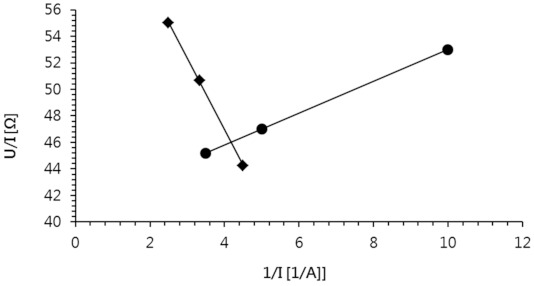
Determination of the limit current.

In order to determine the limiting current density below which the ED process had to be carried out, a U/I = f(1/I) curve was drawn (Figure 3). The limiting current (*I_lim_*) determined from the intersection of two curves was 0.24 A, and the limiting current density (*i_lim_*) could be easily calculated from the formula:

(1) $$i_{lim}=\frac{I_{lim}}{s}$$

where *s* is the membrane effective area, m^2^.

The obtained value of limiting current density was 37.47 A/m^2^.

During the ED experiments, samples of both circulated solutions were obtained at the same time intervals and the conductivity of each sample was measured using a benchtop conductivity meter (Oakton pH/Con 510 Series, EUTECH Instruments, Vernon Hills, IL, USA). The ion concentrations in the diluate and concentrate were determined via ion chromatography (Dionex 2000i/SP, Thermo Fisher Scientific, Waltham, MA, USA) with an anion-exchange column (Dionex Ion Pac AS9HC) with a pre-column (Ion Pac AG9HC) and a cation-exchange column (Ion Pac CS12) protected by a pre-column (CG12A). The total organic carbon (TOC) analysis of non-radioactive solutions was performed using the multi-N/C 3100 apparatus (Analytik Jena AG, Jena, Germany). The TOC content was determined using a carbon analyzer equipped with a nondispersive infrared (NDIR) detector. For samples containing radioactive species, the TOC was measured via a spectroscopic method using NANOCOLOR^®^ OWO 25 and NANOCOLOR^®^ OWO 600 tests. 

The total concentrations of radionuclides in the diluate and concentrate samples were determined using a gamma counter (LG-1b type, INCT, Warsaw, Poland), while the concentrations of specific radionuclides were measured using an automatic gamma counter (PerkinElmer 2480 Wizard2©, Waltham, MA, USA). For each experiment, the reported results were calculated as the mean of at least three independent samples (i.e., all radiometric measurements of each sample were repeated a minimum of three times).

## 3. Results

### 3.1. Experiments Using Model Solutions 

The first experiments were conducted using non-radioactive model solutions that were prepared based on the chemical composition of radioactive liquid waste collected at the RWMP. The types of organic compounds present in this waste were not specified; only their content was known (measured as the TOC). The TOC in different waste samples collected from the RWMP ranged from 15 to 75 mg/dm^3^. Therefore, when preparing the model solutions, it was assumed that both ionic and non-ionic organic compounds were present. In this work, citric acid and octylphenyl ethoxylate (Triton X-102) were added as the model organic substances, which may be present in waste containing decontamination agents from cleaning procedures. Detergents of Triton X-type (e.g., Triton X-100) are also used as a scintillation cocktail in beta scintillation counters, so they may be present in liquid waste originating from radioisotope applications. Four series of solutions, differing in salinity and type of organic substance admixture, were prepared according to Table 2 and employed as model liquid waste in ED experiments.

The preliminary study focused on evaluating the effects of the initial salt concentration of the feed solution and the applied current on the process’s performance. Two current density values were used for each solution: specifically, 18.8 and 35.9 A/m^2^, which were 50% and 96% of the limiting current (for 70% desalination), respectively. The results of these tests are presented in Table 3 and Table 4.

Electrodialysis conducted at a high current was more efficient than the process at a lower amperage (Table 3). Moreover, the results indicated that the total salinity of the solution affected the efficiency of the electrolysis. At lower salt concentrations (solution No. II), a higher desalination of the model solutions was achieved compared with the process applied to solutions with a higher salt concentration. In general, the higher the amperage, the more efficient the removal of citric acid, which is expressed as a decreasing TOC. When a current density of 35.9 A/m^2^ was applied, the TOC decreased by almost 90% in the case of solutions No. I and II. In contrast, in the presence of a lower current density (18.8 A/m^2^), the TOC diminished by 71% and 80% for solutions No. I and II, respectively. The model organic compound (citric acid) added to solutions No. I and II also passed through the membranes, so during the process it was removed from the diluate reservoir and collected in the concentrate container. However, the citrate anions were probably transported from the diluate to the concentrate stream more slowly compared to the other ions, as indicated by lower degree of TOC removal (approximately 85–90%), while the other ions were removed with an efficiency of close to 100%. The transport of ionic organic compounds through the membranes during ED can be influenced by various parameters, such as current density, size, and type of ion charge, as well as the functional groups on these ions [18]. It has been reported that mono-selective anion-exchange membranes can be used to improve the separation of anionic organic substances from salts [19]. However, the separation of ionic organic substances from inorganic compounds present in the model solutions was not observed with the membranes and process conditions applied in this work.

A different situation emerged when Triton X-102 was used as the model non-ionic substance added to the solution (solutions No. III and IV). In this case, the organic substance was retained by the membranes and only a small amount of Triton X-102 passed to the concentrate stream (Table 4).

This experiment demonstrated the suitability of ED for the separation of ionic substances from non-ionic components in solution. These results demonstrated the possibility of selectively separating non-ionic organic contaminants of radioactive liquid waste (which interfere with numerous processes) to treat such waste in the subsequent stages of predisposal. This valuable feature of ED has been used in many fields [20,21,22]. 

### 3.2. Treatment of Radioactive Solutions Using Electrodialysis 

One of the main objectives of this work was to assess the possibility of (i) using ED to remove radionuclides from liquid waste, (ii) concentrating them in a small volume, and (iii) simultaneously obtaining a stream of purified water that can be safely discharged into the environment. For this purpose, two model solutions containing inorganic salts and one of two radionuclides commonly present in real waste collected from Polish laboratories (^137^Cs and ^60^Co) were prepared and subjected to the ED process. The compositions of the model radioactive solutions are presented in Table 5. 

**Figure 4:**
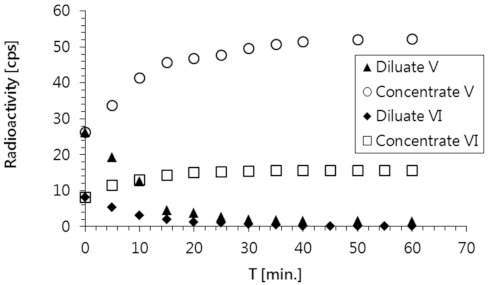
Changes in the radioactivity of the diluate and concentrate streams during the electrodialysis treatment of solutions No. V and VI.

**Figure 5:**
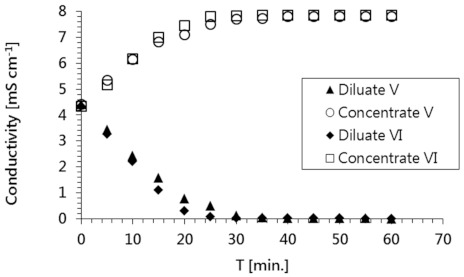
Change in the conductivity of the diluate and concentrate streams during the electrodialysis treatment of solutions No. V and VI.

The electrodialysis was performed using the process parameters selected during the preliminary tests, which provided the best salt removal effects: the voltage (U) was 20 V, the mean velocities (u) of the diluate and concentrate were the same at 0.16 cm/s, and the current density (i) was 31.3 A/m^2^. The results of these experiments are presented in Figure 4 and Figure 5.

These experimental results confirmed that the tested solutions containing radioactive substances were purified with very high efficiency using the applied ED process. Both radionuclides (i.e., ^137^Cs in solution No. V, and ^60^Co in solution No. VI) were completely removed in 40 min (Figure 4). In the same amount of time, a corresponding concentration of these species was detected in the concentrate stream. The efficiency of a radioactive waste purification process is typically described by the decontamination factor (DF), which is calculated using Equation (2),

(2) $$\mathrm{DF}=\frac{A_{0}}{A_{i}}$$

where *A*_0_ and *A_i_* represent the radioactivity of the solution at the end of the experiment and at a given time, respectively (in counts per minute, cpm).

**Figure 6:**
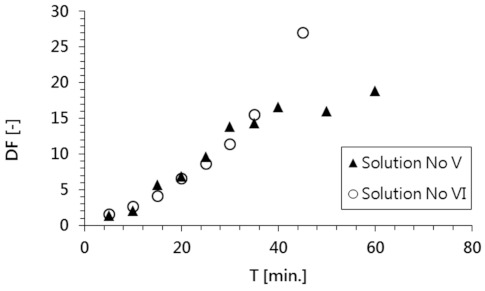
Changes in the decontamination factor during the electrodialysis treatment of solutions No. V and VI.

A comparison of the changes in the decontamination factor of both model solutions is shown in Figure 6. High DF values (19 and 27 for solutions No. V and VI, respectively) were achieved as a result of the electrodialysis process used to decontaminate both model solutions. 

The remaining components in the model solutions (i.e., inorganic salt ions) accumulated in the concentrate, leaving the diluate free of impurities (Figure 5). The decline in conductivity observed for the diluate stream was slightly faster than the decline in this stream’s radioactivity; the diluate attained a conductivity close to that of pure water in just 30 min. Similar behavior was observed for both model radioactive solutions.

Finally, a sample of real waste received from the RWMP was subjected to the electrodialysis treatment process. Its characteristics are summarized in Table 6. 

**Figure 7:**
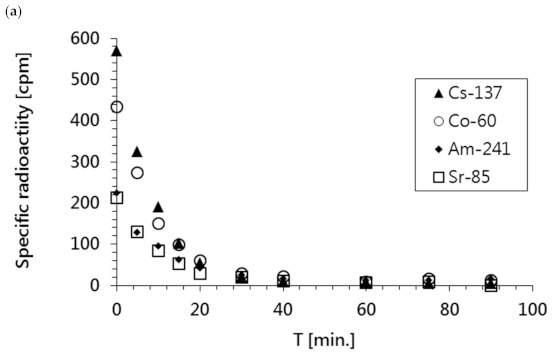

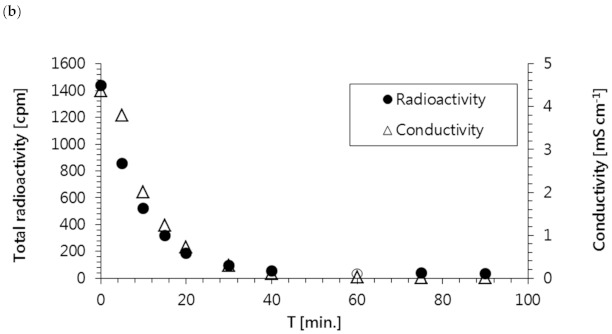
Changes in the radioactivity of the diluate stream during the electrodialysis of the real waste sample. (**a**) Radioactivities of main radioisotopes; (**b**) total radioactivity.

The sample contained ^137^Cs, ^60^Co, ^85^Sr, and ^241^Am radionuclides. The parameters of the applied electrodialysis process were the same as those applied for the treatment of model radioactive solutions. The results of these experiments are presented in Figure 7a,b. 

As shown in Figure 7a, the specific radioactivity of all radionuclides in the waste sample rapidly decreased over time; this was confirmed by the reduction in total activity shown in Figure 7b. Additionally, it was observed that the radioactivities of all radioisotopes and the concentrations of ions present in the solution (represented as conductivity) dropped to the levels of pure water within the first 60 min of the ED process.

In summary, treating the real waste sample using the developed ED process led to efficient salt and radionuclide removal (nearly 100%) after 1 h of treatment, as shown in Table 7.

The content of organic compounds in the final solution (diluate) also diminished. However, at the same time it was observed that organic matter did not accumulate in the concentrate stream—the concentration of organic substances in the concentrate stream slightly decreased as well. This can be explained by membrane fouling caused by organic substances such as ionic surfactants. There are many reports in the literature on the occurrence of membrane fouling during the electrodilysis process of solutions containing surfactants, peptides, or humates [23,24]. They can accumulate on the membrane surface and damage the membrane. This phenomenon occurs mainly when solutions of high concentration are treated or at the end of experiments when a high concentration of electrolyte appears near the membrane surface. It also seems possible to pass the components of the diluate and concentrate into the electrode rinsing solution. However, further research is required to explain the behavior of the organic compounds present in the sample of radioactive wastewater under examination. 

The fouling of the ion exchange membranes is one of the main limiting factors for wide ED implementation, as for other membrane processes used for industrial wastewater treatment. Before implementing the process for the treatment of liquid radioactive waste on an industrial scale, the risk of membrane fouling will certainly require consideration, and the phenomenon itself will necessitate further experiments.

However, we already know many ways to deal with such problems—e.g., by adjusting the hydrodynamic conditions in the apparatus, adding appropriate substances to minimize membrane fouling [25,26], or periodic membrane cleaning. In the described case, fouling can be prevented by adding a small amount of acid (e.g., nitric acid) to the concentrate or by subjecting the wastewater to a preliminary treatment that consists of removing non-ionizable substances (silica, bacteria, soluble organic compounds, etc.).

Overall, the results obtained in this study regarding the treatment of model radioactive solutions and real radioactive waste indicated that the developed ED process (Table 8) could be applied to effectively remove radionuclides as well as inorganic salts present in solutions. Very high levels of salt removal (>99.5%) and high DF values were obtained as a result of this process. 

During the experiments, no significant decrease in the efficiency of the process was observed. However, membrane fouling and scaling can occur during the treatment of real radioactive wastewater by the ED, especially in the case of a long-term process. The membrane supplier predicts the lifetime of the membranes to be 1–3 years. If fouling occurs, cleaning with 5–10% HCl or 4% NaOH can be applied for membrane regeneration. Moreover, the mechanical cleaning of the membrane surfaces should also be carried out periodically.

Certainly, in the further stages of the development of the ED process for the treatment of specific real radioactive wastewater, it will be necessary to carry out longer test cycles to draw final conclusions about the feasibility of the process and to design an industrial system with an electrodialysis chamber.

## 4. Conclusions

Herein, the possibility of using electrodialysis to treat low-level radioactive liquid waste was explored. Such wastewater may contain various types of organic compounds, so the ED process was performed on model solutions containing organic substances (ionic and non-ionic). The results of this study confirmed that ED can be applied for the separation of both organic and inorganic contaminants. Further research indicated that the decontamination of model solutions containing radioactive substances using the ED process was very effective, and all other components of the solutions (i.e., inorganic ions) accumulated in the concentrate, leaving the diluate free from impurities. 

Tests conducted on a real radioactive waste sample showed that the developed ED process was also highly effective for the purification of this waste. Very high levels of salt removal and high decontamination factors were observed.

Based on the results presented in this research, it can be concluded that electrodialysis represents a suitable method for radioactive liquid waste treatment because it ensures high desalination rates and a sufficiently high removal of radioactive substances. If the radioactive liquid waste contains non-ionic organic compounds, their processing via ED using standard cation- and anion-exchange membranes is not limited to the concentration of all substances in the concentrate stream and it is possible to obtain clean water, which can then be used as process water or discharged into the environment. Additionally, the separation of organic compounds from inorganic substances is possible. Overall, this process enables the removal of troublesome components that hinder waste solidification and disposal and allows for the recovery of valuable organic components, if any, from the diluate circuit.

## Figures and Tables

**Table 1:** Parameters of the experimental membranes.

Parameter	PC-SK	PC-SA
Membrane type	Cation-exchange	Anion-exchange
Size, mm	110 × 110	
Active area, cm^2^	64	
Thickness, μm	160–200	180–220
Functional group	–SO_3_^−^	–NR_3_^+^
Resistance, Ω cm^2^	~2.5	~1.8
Water content, wt%	~9	~14

**Table 2:** Schematic composition of model solutions.

	No I	No II	No III	No IV
κ, mS cm^−1^	4.5	2.6	5.5	2.3
Organic substance	Citric acid	Citric acid	Triton X-102	Triton X-102

**Table 3:** The treatment of model solutions containing citric acid using ED.

Compound	Solution No I			Solution No II		
	Initial	Final		Initial	Final	
		18.8 A/m^2^	35.9 A/m^2^		18.8 A/m^2^	35.9 A/m^2^
Na^+^, mg dm^−3^	435.05	130.64	1.69	330.75	0.61	1.09
K^+^, mg dm^−3^	386.6	54.18	0.55	92.5	0.16	0.15
Cs^+^, mg dm^−3^	10.85	2.37	0.00	5.5	0.00	0.01
Mg^2+^, mg dm^−3^	96.55	4.20	0.17	53.1	0.07	0.17
Ca^2+^, mg dm^−3^	87.6	1.59	0.15	-	-	-
Cl^−^, mg dm^−3^	451.75	131.70	1.24	96.15	0.24	0.27
NO_3_^−^, mg dm^−3^	2035.90	341.75	4.49	928	1.62	2.50
SO_4_^2−^, mg dm^−3^	-	-	-	183.35	0.29	0.56
Citric acid, mg dm^−3^	54.45	n.a.	n.a.	75.60	n.a.	n.a.
TOC, mg dm^−3^	29.2	4.20	3.02	14.7	2.07	2.19

**Table 4:** The treatment of model solutions containing Triton X-102 using ED.

Compound	Solution No III		Solution No IV	
	Initial	Final	Initial	Final
Na^+^, mg dm^−3^	160.12	2.92	121.52	1.37
K^+^, mg dm^−3^	379.56	5.83	94.91	0.00
Cs^+^, mg dm^−3^	84.90	0.00	50.75	0.00
Mg^2+^, mg dm^−3^	120.33	12.50	64.49	3.26
Ca^2+^, mg dm^−3^	374.85	32.56	-	-
Cl^−^, mg dm^−3^	339.41	2.68	65.66	0.00
NO_3_^−^, mg dm^−3^	1784.37	78.21	925.41	76.97
SO_4_^2−^, mg dm^−3^	-	-	141.42	0.00
Triton X-102, mg dm^−3^	49.0	n.a.	33.0	n.a.
TOC, mg dm^−3^	30.91	27.83	18.02	17.84

**Table 5:** Characteristics of the radioactive model solutions.

Component	Solution No V	Solution No VI
^137^Cs, cps *	26.3	-
^60^Co, cps *	-	8.1
MgCl_2_, mg dm^−3^	402.3	
CaCl_2_, mg dm^−3^	477.4	
Na_2_SO_4_, mg dm^−3^	667.5	
KNO_3_, mg dm^−3^	854.6	
NaNO_3_, mg dm^−3^	1700	
κ, mS cm^−1^	4.41	4.33

* cps—counts per second.

**Table 6:** Composition of the radioactive liquid waste sample.

Parameter	Quantity
^137^Cs, cpm *	569.3
^60^Co, cpm *	433.0
^85^Sr, cpm *	213.4
^241^Am, cpm *	224.1
Cl^−^, mg dm^−3^	26.6
NO_3_^−^, g dm^−3^	1.8
SO_4_^2−^, mg dm^−3^	451.3
Na^+^, mg dm^−3^	635.5
K^+^, mg dm^−3^	33.0
Mg^2+^, mg dm^−3^	102.7
Ca^2+^, mg dm^−3^	172.2
TOC, mg dm^−3^	18.4
pH	5.0
κ, mS cm^−1^	4.3

* cpm—counts per minutes.

**Table 7:** The treatment of liquid waste sample using ED.

Parameter	Initial	After ED
Total radioactivity, cpm including:	1448.9	32.4
^137^Cs, cpm	569.3	3.9
^60^Co, cpm	433.0	12.9
^85^Sr, cpm	213.4	0
^241^Am, cpm	224.1	15.6
κ, mS cm^−1^	4.3	0.016
TOC, mg dm^−^^3^	18.4	12.2

**Table 8:** Summary of the results from treating model solutions and real waste using the developed ED process.

	Decontamination Factor (DF)	Salt Removal (SR)
Solution No V	18.89	99.86%
Solution No VI	27.00	99.86%
Sample of real waste	44.42	99.63%

## Data Availability

Not applicable.
